# Spotlights on adult patients with pediatric-type diffuse gliomas in accordance with the 2021 WHO classification of CNS tumors

**DOI:** 10.3389/fnins.2023.1144559

**Published:** 2023-05-05

**Authors:** Wenlin Chen, Shanmu Jin, Qianshu Liu, Hai Wang, Yu Xia, Xiaopeng Guo, Siying Guo, Yaning Wang, Yixin Shi, Delin Liu, Yilin Li, Yuekun Wang, Hao Xing, Junlin Li, Jiaming Wu, Tingyu Liang, Tian Qu, Huanzhang Li, Tianrui Yang, Kun Zhang, Yu Wang, Wenbin Ma

**Affiliations:** ^1^Department of Neurosurgery, Center for Malignant Brain Tumors, National Glioma MDT Alliance, Peking Union Medical College Hospital, Chinese Academy of Medical Sciences and Peking Union Medical College, Beijing, China; ^2^‘4+4’ Medical Doctor Program, Chinese Academy of Medical Sciences and Peking Union Medical College, Beijing, China; ^3^Eight-Year Medical Doctor Program, Chinese Academy of Medical Sciences and Peking Union Medical College, Beijing, China; ^4^China Anti-Cancer Association Specialty Committee of Glioma, Beijing, China

**Keywords:** glioma, 2021 WHO classification of central nervous system tumors, pediatric-type diffuse gliomas, adult patients, molecular pathology

## Abstract

**Introduction:**

The fifth edition of the World Health Organization (WHO) classification of central nervous system (CNS) tumors released in 2021 formally defines pediatric-type diffuse gliomas. However, there is still little understanding of pediatric-type diffuse gliomas, and even less attention has been paid to adult patients. Therefore, this study describes the clinical radiological, survival, and molecular features of adult patients with pediatric-type glioma.

**Methods:**

Adult patients who underwent surgery from January 2011 to January 2022, classified as pediatric-type glioma, were included in this study. Clinical, radiological, histopathological, molecular pathological, and survival data were collected for analysis.

**Results:**

Among 596 adult patients, 20 patients with pediatric-type glioma were screened, including 6 with diffuse astrocytoma, *MYB*- or *MYBL1*-altered, 2 with diffuse midline glioma, H3 K27-altered, and 12 with diffuse pediatric-type high-grade glioma, H3-wildtype and IDH-wildtype. Pediatric high-grade glioma (pHGG) frequently showed tumor enhancement, peritumoral edema, and intratumoral necrosis. Adult patients with pHGG showed a longer life expectancy than adult patients with glioblastoma. Common molecular alterations included chromosome alterations and *CDKN2A/B*, *PIK3CA*, and *PTEN*, while altered *KMT5B* and *MET* were found to affect the overall survival.

**Conclusion:**

Our study demonstrated adult patients with pediatric-type glioma. Notably, our research aims to expand the current understanding of adult patients with pediatric-type diffuse gliomas. Furthermore, personalized therapies consisting of targeted molecular inhibitors for *MET* and *VEGFA* may exhibit beneficial effects in the corresponding population.

## Introduction

Diffuse gliomas, known as a group of astrocytic and oligodendroglial tumors with infiltrative growth patterns, predominate in malignant primary brain tumors and occur across all age groups, with an average annual incidence of 0.47, 1.84, and 8.72 per 100,000 in children, adolescents and young adults (AYA), and older adults (Ostrom et al., 2022). Even with the combined treatment of surgery, radiotherapy, chemotherapy, targeted therapy, and tumor treating fields, the overall prognosis remains dismal (Stupp et al., 2017). Given the poor prognosis status and intertumoral heterogeneity, researchers have been striving for a classification criterion to more precisely stratify treatment response and survival outcome. The 2016 World Health Organization (WHO) classification of central nervous system (CNS) tumors is based mainly on histology (Louis et al., 2016), while several prognostic molecular markers have been identified in recent years. Therefore, integrating molecular pathologic features into the 2021 WHO classification of CNS tumors updates our understanding of diffuse gliomas (Louis et al., 2021). For instance, IDH-wildtype diffuse astrocytoma or anaplastic astrocytoma with *EGFR* amplification, combined whole chromosome 7 gain and whole chromosome 10 loss (+7/−10) or *TERT* promoter mutation, resembles IDH-wildtype glioblastoma in the aggressive clinical course, underlining the importance of combining histopathology and molecular pathology in diffuse glioma diagnosis (Brat et al., 2018).

Pediatric-type diffuse gliomas, whose molecular alterations and survival outcomes are quite different from adult-type counterparts (Jones et al., 2012), are formally recognized in the latest classification and divided into pediatric low-grade glioma (pLGG) and pediatric high-grade glioma (pHGG) (Louis et al., 2021). pLGGs include diffuse astrocytoma, *MYB*- or *MYBL1*-altered; angiocentric glioma; polymorphous low-grade neuroepithelial tumor of the young; and diffuse low-grade glioma, MAPK pathway-altered. pHGGs include diffuse midline glioma (DMG), H3 K27-altered; diffuse hemispheric glioma, H3 G34-mutant; diffuse pediatric-type high-grade glioma, H3-wildtype and IDH-wildtype; and infant-type hemispheric glioma. Besides, the denotations of adult-type and pediatric-type are not strictly limited to the population of the corresponding age stratum, giving us a hint on the classification of diffuse gliomas in adult patients. Adult cases of diffuse astrocytoma or anaplastic astrocytoma, IDH-wildtype failing to fall into glioblastoma, IDH-wildtype for lack of *EGFR* amplification, +7/−10 and *TERT* promoter mutation, should undergo additional molecular tests (e.g., *MYB*, *MYBL1*, *BRAF*, *FGFR1*, histone H3) and may be classified as certain subtype of pediatric-type diffuse glioma (Gritsch et al., 2022). Under the new classification, there is still a lack of clinical description of the pediatric type of diffuse glioma (Hong et al., 2022; Kalelioglu et al., 2022), and even less attention has been paid to adult patients in the pediatric type. Additionally, previous studies have tended to classify such patients as adult-type patients, with implications for both prognostic estimation and treatment. However, investigating adult patients with pediatric-type diffuse gliomas is essential for discriminating them from adult-type in clinical settings.

This research focused on and comprehensively analyzed the histological and molecular data of 20 adult patients with pediatric-type glioma according to the 2021 WHO classification of CNS tumors. Further studies were conducted to contribute preliminary knowledge regarding this population. Notably, we described their basic clinical information, radiological features, and molecular landscape. Likewise, the overall survival and possible prognostic molecular alterations were discussed.

## Methods

### Study population

Patients who underwent surgery at the Department of Neurosurgery at Peking Union Medical College Hospital (PUMCH) from January 2011 to January 2022 were screened (Guo et al., 2023). Adult patients (≥18 years old) classified as pediatric-type glioma according to the 2021 WHO classification of CNS tumors were enrolled in this study. Among them, patients with comprehensive clinical data were included for further analysis. The study was approved by the Institutional Ethics Review Board of PUMCH (S-424) and conformed to the requirements of the Declaration of Helsinki.

### Clinical and radiological data collection

Clinical and radiological data were collected retrospectively from the medical records. Clinical information collected for analysis included gender, age of diagnosis, course of the disease, baseline Karnofsky performance status (KPS) score, clinical symptoms, the extent of surgical resection (ESR), and postoperative treatments. The survival status was collected through outpatient and telephone follow-ups. Overall survival (OS) was defined as the time from surgery to the patient’s death or final follow-up (treated as censored values). Furthermore, median overall survival was defined as the overall survival achieved by 50% of included patients.

Radiological features were collected from the baseline magnetic resonance imaging (MRI), including tumor number, tumor location, involvement of functional areas, maximum diameter of tumor, maximum diameter of peritumoral edema, cyst cavity, necrotic centers, and tumor appearance on T1WI, T2WI, and T1-enhanced sequences. The evaluation of images was performed by a team of radiologists, including 2 junior residents responsible for specific feature extraction, and 1 chief radiologist responsible for re-examination of the results. All radiologists have board certification.

### Tumor pathology data collection

Tumor pathology classification was determined with the 2021 WHO classification of CNS tumor criteria, and histopathological and molecular pathological data were collected. Histopathological data were obtained from the pathological reports by PUMCH, mainly including the Ki-67 index and histological grade. For molecular pathology, we screened 60 molecular markers, including *IDH1/2*, *MYB*, *MYBL1*, *EGFR*, and *CDKN2A/B*, summarized from recent studies on tumorigenesis and prognosis of glioma. We analyzed the molecular alterations of each enrolled patient using next-generation sequencing (NGS), polymerase chain reaction (PCR)-based assays, and fluorescence *in situ* hybridization methods (FISH). The list of molecular markers included in this study is shown in Supplementary Table 1. Furthermore, we demonstrated the features of adult patients with each subgroup of pediatric-type glioma in terms of clinical information, radiological features, prognostic status, and molecular alterations.

### Statistical analysis

Normally distributed variables were compared by Student’s *t*-test, and non-normally distributed variables were compared between groups by Kruskal-Wallis *H* test. Comparisons of categorical variables were performed by the chi-squared test. Additionally, survival analyses were performed using the Kaplan–Meier method and log-rank test and were presented by the Kaplan–Meier curves. *p* < 0.05 was considered to be statistically significant. All statistical analyses were performed with SPSS (version 26.0, IBM, USA) statistical software, and graphs were created using R Studio (PBC & Certified B Corp.^®^, USA) and GraphPad Prism (9, GraphPad Software, USA) software.

## Results

### Baseline information of adult patients with pediatric-type diffuse gliomas

Among 596 adult patients, 20 patients with pediatric-type glioma were included in this study. Of these patients, 6 presented diffuse astrocytoma, *MYB*- or *MYBL1*-altered (all previously diagnosed as diffuse astrocytoma, IDH-wildtype), 2 with diffuse midline glioma, H3 K27-altered (both previously diagnosed as diffuse midline glioma, H3 K27M-mutant), and 12 with diffuse pediatric-type high-grade glioma, H3-wildtype and IDH-wildtype (all previously diagnosed as anaplastic astrocytoma, IDH-wildtype).

Among the 20 patients enrolled, 13 were male, 7 were female, the mean age of diagnosis was 46 years. The median preoperative KPS of enrolled patients was 92.5. Regarding clinical symptoms, intracranial hypertension, neurological deficits, and epilepsy occurred in 5, 12, and 2 patients, respectively. For ESR, all patients experienced surgery, 55.0% (11/20) underwent gross total resection, 15.0% (3/20) went through subtotal resection, and 30.0% (6/20) received tumor biopsy only. For comprehensive postoperative therapy, 3 patients received the standard Stupp regimen (Stupp et al., 2009) (radiotherapy with concomitant temozolomide followed by up to six cycles of adjuvant temozolomide), while 7 did not. Furthermore, 10 patients were unable to be classified due to incomplete records. The specific information is shown in Table 1.

**Table 1:** Basic clinical characteristics of adult patients with pediatric-type diffuse gliomas.

	All Patients (*n* = 20)	Diffuse pediatric-type high-grade glioma, H3- and IDH-wildtype (*n* = 12)	Diffuse astrocytoma, *MYB*- or *MYBL1*-altered (*n* = 6)	Diffuse midline glioma, H3 K27-altered (*n* = 2)
Male, n/%	13, 65.0%	7, 58.3%	4, 66.7%	2, 100%
Age, years	46 ± 11.5	49 ± 10.6	44 ± 10.7	45, 25
Age ≥ 45, n/%	12, 60.0%	7, 58.3%	4, 66.7%	1, 50%
Age ≥ 60, n/%	2, 10.0%	2, 16.7%	0, 0.0%	0, 0%
Disease duration before admission, weeks	9 (3, 16)	12.5 (4.75, 38)	9.5 (3, 16)	3, 3
Baseline KPS score	92.5 (70, 95)	85 (80, 95)	95 (70, 95)	70, 100
Intracranial hypertension, n/%	5, 25.0%	2, 16.7%	2, 33.3%	1, 50%
Epilepsy, n/%	2, 10.0%	1, 8.3%	1, 16.7%	0, 0%
Neurologic impairment, n/%	12, 60.0%	8, 66.7%	3, 50.0%	1, 50%
Motor dysfunction, n/%	8, 40.0%	5, 41.7%	2, 33.3%	1, 50%
Sensory dysfunction, n/%	5, 20.0%	3, 25.0%	1, 16.7%	1, 50%
Visual field defect, n/%	1, 5.0%	1, 8.3%	0, 0%	0, 0%
Aphasia, n/%	1, 5.0%	1, 8.3%	0, 0%	0, 0%
Extent of surgical resection				
Gross total resection, n/%	11, 55.0%	7, 58.3%	3, 50.0%	1, 50%
Subtotal resection, n/%	3, 15.0%	1, 8.3%	1, 16.7%	1, 50%
Biopsy, n/%	6, 30.0%	4, 33.3%	2, 33.3%	0, 0%
Post-operative treatment				
Stupp protocol w/o other therapies, n/%	3, 30%	3, 42.9%	0, 0.0%	0, 0.0%
Without standardized Stupp protocol, n/%	7, 70%	4, 57.1%	2, 100.0%	1, 100%
NA, n	10	5	4	1

Basic clinical characteristics of all adult patients with pediatric-type diffuse glioma (*n* = 20) including diffuse pediatric-type high-grade glioma, H3- and IDH-wildtype (*n* = 12), diffuse astrocytoma, *MYB*- or *MYBL1*-altered (*n* = 6) and diffuse midline glioma, H3 K27-altered (*n* = 2). KPS, Karnofsky Performance Status; NA, not available; w/o, with or without.

### Radiological features of adult patients with pediatric-type diffuse gliomas

**Figure 1:**
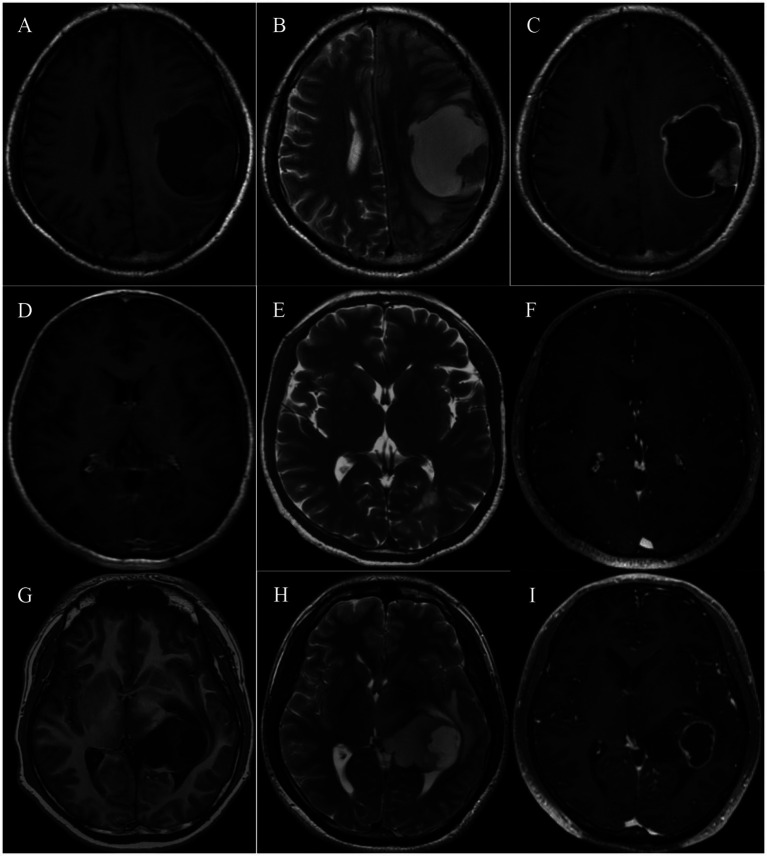
Typical MRI imaging of pediatric-type diffuse gliomas in adults. **(A–C)** MRI of a 32-year-old patient with primary diffuse pediatric-type high-grade glioma, H3-wildtype and IDH-wildtype located in the left frontal and parietal lobe. The lesion shows a mixed signal on T1 and T2-weighted imaging and consists of an intermediately enhancing nodule and a ring-like enhancing cyst cavity. **(D–F)** MRI of a 47-year-old patient with diffuse astrocytoma, *MYB*- or *MYBL1*-altered, located in the left occipital lobe. The restricted lesion is hypointense on T1-weighted imaging, hyperintense on T2-weighted imaging, and not enhanced. **(G–I)** MRI of a 25-year-old patient with diffuse midline glioma, H3 K27-altered, located in the left thalamus and lateral ventricle. The cystic-solid lesion shows a mixed signal on T1 and T2-weighted imaging and ring-like enhancement.

Neurological imaging is a crucial basis of diagnosis for intracranial tumors, and we summarized the radiological characteristics of patients with pediatric-type diffuse gliomas. After removing missing data, 6 patients with diffuse astrocytoma, *MYB*- or *MYBL1*-altered, 1 with diffuse midline glioma, H3 K27-altered, and 7 with diffuse pediatric-type high-grade glioma, H3-wildtype and IDH-wildtype were included in the analysis, and representative radiological images are shown (Figure 1).

In terms of tumor distribution, it was prevalent for patients to have single tumors across different groups, and involvement of functional areas was relatively rare. The mean maximum tumor diameter was 4.19, 4.96, and 4.17 cm for diffuse astrocytoma, *MYB*- or *MYBL1*-altered, diffuse midline glioma, H3 K27-altered, and diffuse pediatric-type high-grade glioma, H3-wildtype and IDH-wildtype, respectively. MRI in all groups generally showed low or mixed density on T1W1 and high or mixed density on T2W1. On T1-enhanced sequence, 6/7 (85.7%) patients with diffuse pediatric-type high-grade glioma, H3-wildtype and IDH-wildtype tended to exhibit ring-like and heterogeneous enhancement. In contrast, fewer patients (3/6, 50%) with diffuse astrocytoma, *MYB*- or *MYBL1*-altered, showed enhancement. Patients with high-grade pediatric-type glioma showed a higher incidence of peritumoral edema (85.7% vs. 50%) and intratumoral necrosis (71.4% vs. 16.7%) but relatively smaller diameter in both radiological features (2.72 cm vs. 3.39 cm; 2.38 cm vs. 4.99 cm). Table 2 summarizes the comprehensive radiological features of adult patients with pediatric-type diffuse glioma.

**Table 2:** Radiological features of adult patients with pediatric-type diffuse gliomas.

Radiological features	Total (*n* = 14)	Diffuse pediatric-type high-grade glioma, H3-wildtype and IDH-wildtype (*n* = 7)	Diffuse astrocytoma, *MYB*- or *MYBL1*-altered (*n* = 6)	Diffuse midline glioma, H3 K27-altered (*n* = 1)
Number				
1	10 (71.4%)	4 (57.1%)	5 (83.3%)	1 (100%)
2	1 (7.1%)	1 (14.3%)	–	–
3	1 (7.1%)	1 (14.3%)	–	–
Diffuse	2 (14.3%)	1 (14.3%)	1 (16.7%)	–
Side				
Left	7 (50.0%)	1 (14.3%)	5 (83.3%)	1 (100%)
Right	4 (28.6%)	4 (57.1%)	–	–
Bilateral	3 (21.4%)	2 (28.6%)	1 (16.7%)	–
Location				
Single lobe^a^	4 (28.6%)	1 (14.3%)	3 (50.0%)	–
Multiple lobes	3 (21.4%)	2 (28.6%)	1 (16.7%)	–
Supratentorial with midline^b^ involved	5 (35.7%)	2 (28.6%)	2 (33.3%)	1 (100%)
Infratentorial	2 (14.3%)	2 (28.6%)	–	–
Function^c^				
Single function	3 (21.4%)	–	3 (50.0%)	–
Multiple functions	2 (14.3%)	1 (14.3%)	1 (16.7%)	–
None	9 (64.3%)	6 (85.7%)	2 (33.3%)	1 (100%)
T1WI				
Low	9 (64.3%)	4 (57.1%)	5 (83.3%)	–
Equal	–	–	–	–
Mixed	5 (35.7%)	3 (42.9%)	1 (16.7%)	1 (100%)
T2WI				
Equal	1 (7.1%)	–	1 (16.7%)	–
High	8 (57.1%)	4 (57.1%)	4 (66.7%)	–
Mixed	5 (35.7%)	3 (42.9%)	1 (16.7%)	1 (100%)
Contrast-Enhanced	10 (71.4%)	6 (85.7%)	3 (50.0%)	1 (100%)
Ring-like Non-ring-like	6 (60.0%)	3 (50.0%)	2 (66.7%)	1 (100%)
	4 (40.0%)	3 (50.0%)	1 (33.3%)	–
Homogeneous	1 (10.0%)	–	1 (33.3%)	–
Heterogeneous	9 (90.0%)	6 (100%)	2 (66.7%)	1 (100%)
Tumor maximum diameter, cm	4.23 ± 1.94	4.17 ± 2.25	4.19 ± 1.89	4.96
Peritumoral edema	10 (71.4%)	6 (85.7%)	3 (50.0%)	1 (100%)
Edema maximum diameter, cm	2.79 ± 1.67	2.72 ± 1.55	3.39 ± 2.24	1.38
Cyst cavity	4 (28.6%)	2 (28.6%)	1 (16.7%)	1 (100%)
Cystic cavity maximum diameter, cm	4.18 ± 1.65	4.07 ± 2.83	4.54	4.05
Necrotic center	6 (42.9%)	5 (71.4%)	1 (16.7%)	–
Necrotic center maximum diameter, cm	2.82 ± 1.55	2.38 ± 1.27	4.99	–

The data is presented with n (%) or mean ± SD. ^a^Lobes include frontal, parietal, occipital, temporal and insular. ^b^Supratentorial midline structures include the corpus callosum, basal ganglia, thalamus, third ventricle, and midbrain. ^c^Functions include motor, sensor, language, and vision.

### Survival of adult patients with pediatric-type diffuse gliomas

**Figure 2:**
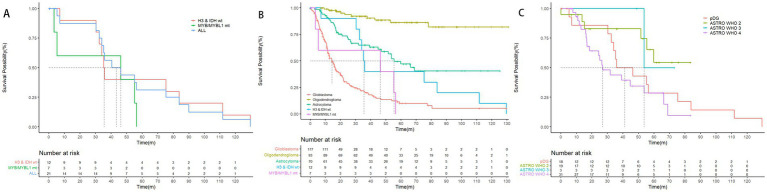
Overall survival of pediatric-type glioma based on the 2021 WHO classification of CNS tumors. **(A)** The mOS of pediatric-type glioma was 43.4 months, mOS of the 2 subtypes, diffuse pediatric-type high-grade glioma, H3-wildtype and IDH-wildtype, and diffuse astrocytoma, *MYB*- or *MYBL1*-altered were 35.5 months, and 46.3 months (*p* = 0.43). **(B)** The mOS of glioblastoma, astrocytoma, diffuse pediatric-type high-grade glioma, H3-wildtype and IDH-wildtype, and diffuse astrocytoma, *MYB*- or *MYBL1*-altered were 14.3 months, 55.4 months, 35.5 months, and 46.3 months. The mOS of oligodendroglioma cannot be calculated, as the survival possibility was above 50% at the time of follow-up. Notice that the mOS of glioblastoma was significantly shorter than diffuse pediatric-type high-grade glioma, H3-wildtype and IDH-wildtype (*p* = 0.047). **(C)** The mOS of diffuse pediatric-type high-grade glioma, H3-wildtype and IDH-wildtype and WHO grade 2, 3, and 4 diffuse astrocytoma were 35.5 months, NA, 53.6 months, and 26.9 months. Although the results did not indicate a statistically significant difference, there was a clear trend that mOS of patients decreased as the WHO grading increased. The mOS of patients with pediatric-type glioma was roughly between that of WHO grade 3 diffuse astrocytoma and WHO grade 4 diffuse astrocytoma.

We further explored the survival of adult patients with pediatric-type diffuse glioma and compared it with adult-type diffuse glioma. The median OS (mOS) of diffuse pediatric-type high-grade glioma, H3-wildtype and IDH-wildtype was 35.5 months. In contrast, the mOS of diffuse astrocytoma, *MYB*- or *MYBL1*-altered, was 46.3 months, suggesting that adult patients with high-grade pediatric-type diffuse glioma are less likely to experience longer survival and better prognosis. Compared with glioblastoma (mOS = 14.3 months) and astrocytoma (mOS = 55.4 months), adult patients with pediatric-type high-grade glioma showed significantly better mOS than adult patients with glioblastoma (*p* = 0.047), while no statistical significance were observed when comparing pediatric-type high-grade glioma and WHO grade 2, 3, 4 astrocytoma. These results and specific survival information are presented in Figure 2.

### Molecular characteristics in adult patients with pediatric-type diffuse gliomas

**Figure 3:**
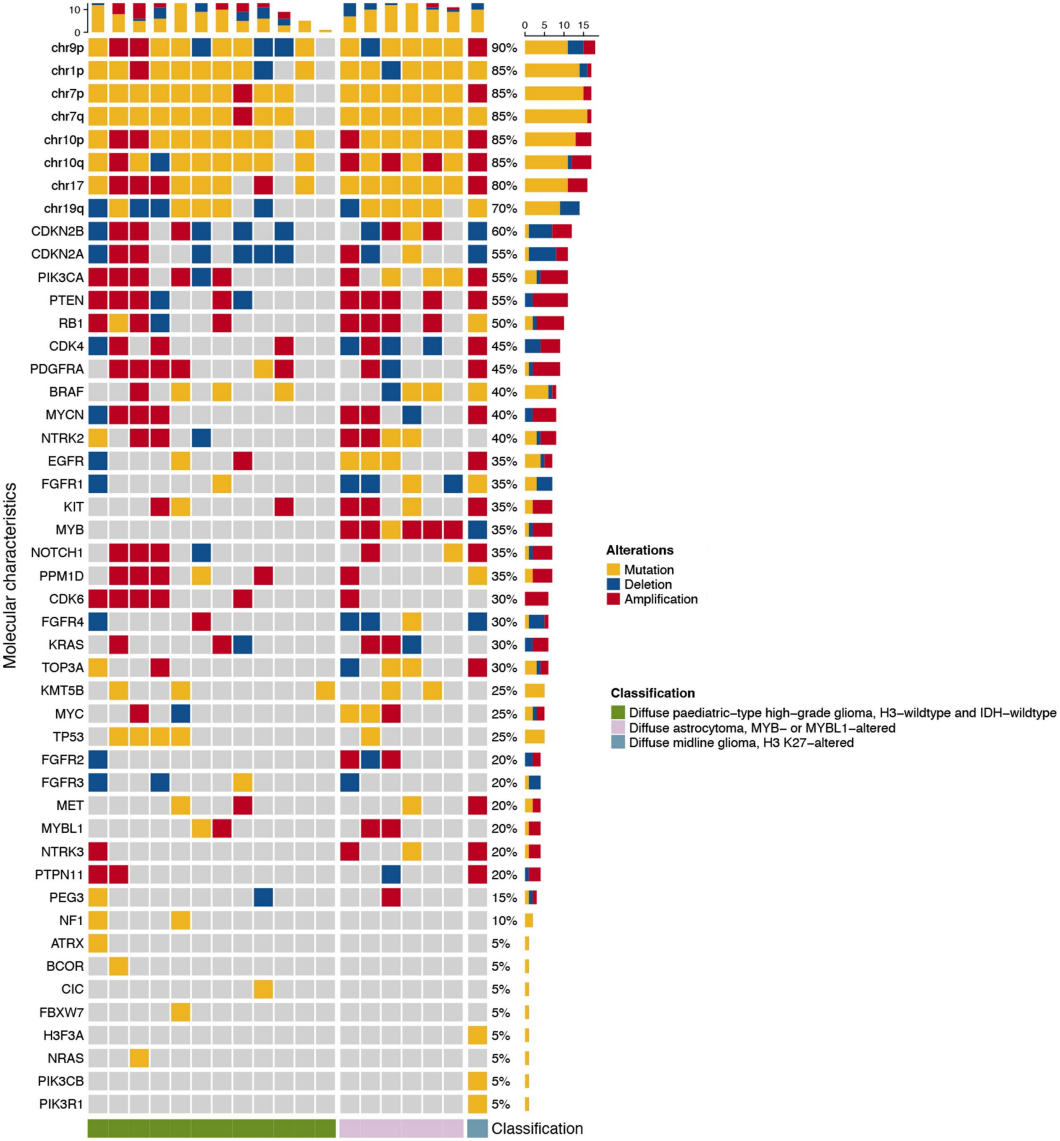
Waterfall heatmap of the molecular alterations of the 19 adult patients with pediatric-type glioma based on the 2021 WHO classification of CNS tumors. Each column represents an individual patient, and the cube’s color indicates the alteration status of each molecular characteristic.

Different subtypes of pediatric-type diffuse glioma showed distinct molecular characteristics, including mutations, amplifications, or deletions of chromosomes and genes. We analyzed the molecular alterations of tumors in each group of adult patients with pediatric-type diffuse gliomas. One patient was excluded because of an unaccomplished molecular pathological examination due to incomplete specimens. Figure 3 shows the alterations of molecular markers for each group of patients. Common genetic alterations in adult patients with pediatric-type diffuse gliomas mainly included chromosome alterations and *CDKN2A/B* (55 and 60%), *PIK3CA* (55%), and *PTEN* (55%).

### Implications of molecular alterations with survival in adult patients with pediatric-type diffuse gliomas

**Figure 4:**
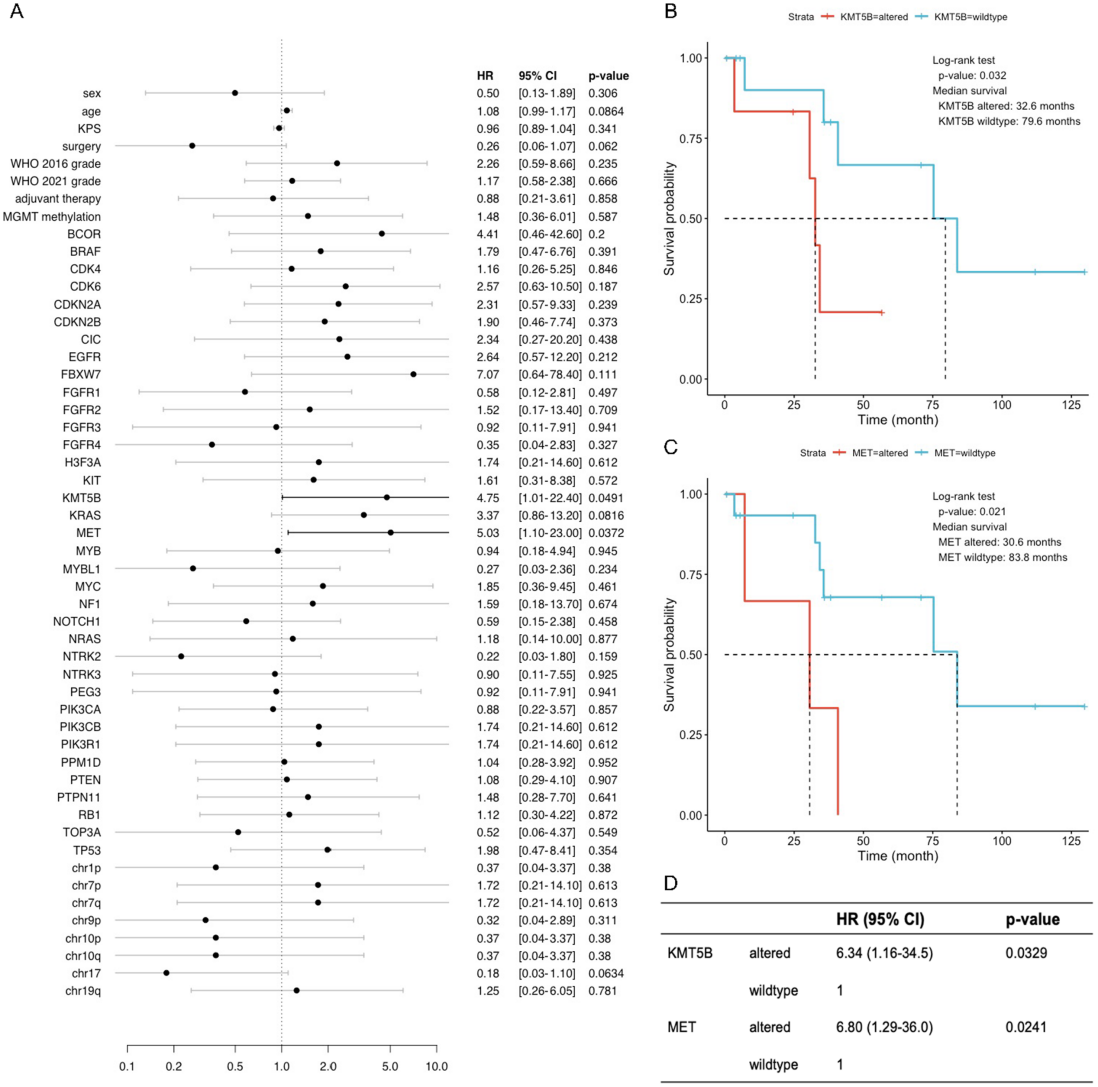
*KMT5B* and *MET* were identified as important prognostic factors for adult patients with pediatric-type glioma. **(A)** Forest plot of univariate Cox regressions of clinical and molecular variables. Only variables with calculable value of *p* and finite confidence intervals are shown. **(B,C)** Kaplan–Meier survival curves of two variables (*KMT5B* and *MET*) with significant results (*p* < 0.05) in univariate Cox analysis. **(D)** Multivariate Cox regression on *KMT5B* and *MET*, indicating that each factor exerts an independent effect on overall survival.

Currently, molecular markers for adult glioma classification and prognosis include *IDH1/2* mutations, homozygous deletion of *CDKN2A* and/or *CDKN2B*, *EGFR* amplification, and *TERT* promoter mutation. This study focused on the molecular pathology of adult patients with pediatric-type diffuse gliomas. Other clinical markers and molecular alterations associated with patient prognosis were also explored to provide clues for prognostic prediction and clinical decision-making. The results of multivariate regression analysis showed that alterations in *KMT5B* and *MET* were associated with shorter overall OS in adult patients with pediatric-type diffuse gliomas, with a value of *p* of 0.032 and 0.021, respectively (Figure 4).

## Discussion

Since the new 2021 WHO classification of CNS tumors (Louis et al., 2021), studies on pediatric-type diffuse gliomas, especially in adult patients, have been lacking. Thus, this study included 20 adult patients with pediatric-type diffuse gliomas of 3 subtypes, and differential clinical features were discovered among these subgroups. For the radiological characteristics, tumor enhancement, peritumoral edema, and intratumoral necrosis were more frequently observed in the pHGG subgroup, corresponding to stronger invasiveness. Notably, adult patients with pHGG showed a relatively longer life expectancy than patients with glioblastoma. Additionally, *CDKN2A/B*, *PIK3CA*, and *PTEN* alterations were commonly observed in adult patients with pediatric-type diffuse gliomas. As for molecular alterations, altered *KMT5B* and *MET* were found to prominently affect overall survival.

In this study, we described and analyzed adult patients with pediatric-type diffuse gliomas, particularly diffuse astrocytoma, *MYB*- or *MYBL1*-altered, diffuse midline glioma, H3 K27-altered, and diffuse pediatric-type high-grade glioma, H3-wildtype and IDH-wildtype. In this cohort, the median age is 46 years with a standard deviation (SD) of >10 years, which differs from the studies that suggested pediatric diffuse glioma occurs predominantly in children and young adults (Schramm et al., 2004). One of the possible reasons may be that our hospital receives mainly adult patients, and the absence of typical clinical symptoms may be related to their older age at diagnosis. At the same time, current researches focused mainly on pediatric cases, and the occurrence in the adult population may be expounded insufficiently. Patients with diffuse astrocytoma, *MYB*- or *MYBL1*-altered are mostly diagnosed with drug-resistant seizures, previous study suggested that 81% of these patients develop epilepsy in childhood and the median age of seizure onset is 10 years (Wefers et al., 2020). H3-wildtype and IDH-wildtype patients typically diagnosed in children, adolescents or young adults, and a meta-analysis of 190 patients found that the median age in this group was less than 12 years, although age of patients was broadly distributed with the oldest age being over 30 years (Mackay et al., 2017). For H3 K27-altered diffuse midline gliomas, the reported age of diagnosis is 30 years for adult patients, which is quite consistent with the patients we included that diagnosed as 45 and 25 years, respectively. More details shown in Supplementary Figure 1 and a review by statistician was accomplished (Meyronet et al., 2017; Dono et al., 2020; Enomoto et al., 2020; Lv et al., 2022). Twelve patients were initially diagnosed as anaplastic astrocytoma, IDH-wildtype, and reclassified as H3-wildtype and IDH-wildtype diffuse pediatric-type high-grade glioma according to the 2021 WHO classification of CNS tumors (Louis et al., 2016). Diffuse pediatric-type high-grade glioma, H3-wildtype and IDH-wildtype can further be divided into 3 subtypes: diffuse pediatric-type high-grade glioma RTK2, diffuse pediatric-type high-grade glioma RTK1, and diffuse pediatric-type high-grade glioma MYCN (Korshunov et al., 2017; Ellison et al., 2019). Notably, H3-wildtype and IDH-wildtype pHGG show a relatively benign course compared to their adult counterparts. A multicenter study indicated a 2-year survival rate of 23.5% and mOS of 17.2 months (Mackay et al., 2017). In this study, survival analysis of different subtypes showed that H3-wildtype and IDH-wildtype pHGG have significantly longer mOS (35.5 months) than glioblastoma IDH-wildtype (14.3 months, *p* = 0.047). Prognostic risk factors for this subtype of pediatric-type glioma are currently unknown. However, a study conducted in 2022 analyzed 17 pediatric patients and concluded that *TP53* mutation is a significant risk factor for poor prognosis (Hong et al., 2022).

Diffuse astrocytoma, *MYB*- or *MYBL1*-altered accounts for approximately 2% of all pediatric low-grade gliomas composed of monomorphic cells. *MYB* is a family of genes containing the *MYB/SANT* structural domain transcription factor, and the *MYBL1* gene plays a similar role (Baker et al., 2009; Kim et al., 2018). Other possible molecular features include *BRAF* and *FGFR1* alterations (Qaddoumi et al., 2016; Barinfeld et al., 2022). Previous reports of this pediatric type of tumor are scarce, especially studies focusing on adult patients. Wefers et al. analyzed the survival of 18 patients (mainly adults). They showed a good prognosis with a median follow-up of 2.5 years and only one recurrence during the follow-up period, which was subsequently eradicated by re-operation (Wefers et al., 2020). In our study, we described a total of 6 patients in terms of clinical, radiological, molecular, and prognostic characteristics, which were generally consistent with previous studies.

Two adult patients with DMGs located in the brain’s midline, involving the temporal lobe, thalamus, and pons (Meyronet et al., 2017). Thalamic DMGs are rarely reported, representing 1–5% of pediatric brain tumors (Mackay et al., 2018; Roux et al., 2020). In terms of clinical manifestations, one patient had prominent characteristics of pyramidal tract injury (reported incidence was 51%), as well as signs of limb sensory abnormalities and intracranial hypertension (Hoffman et al., 2018). For the radiological characteristics, previous researches indicated that DSC-MRI parameters, ADC values, and the T2-FLAIR mismatch sign are valuable in diagnosis and classification of DMGs (Kurokawa et al., 2022). However, due to the small sample size and insufficient MRI sequence, we were unable to verify this conclusion. Notably, the patient was diagnosed as both H3 p.K28M (K27M) mutation (for H3 K27-mutant subtypes) and amplification of *EGFR* (for the *EGFR*-mutant subtype), though it is reported that *EGFR*-altered DMGs most often occur during childhood (Broniscer et al., 2018; Castel et al., 2018).

Generally, pediatric gliomas exhibit hypointensity or isointensity on T1 (McNamara et al., 2022), a finding that was also observed in all samples of our study. In diffuse astrocytoma, *MYB*- or *MYBL1* altered, large cysts are commonly reported, however, in our study, cyst was only observed in one case, albeit with the largest cyst diameter among all samples. Diffuse midline gliomas, H3 K27-altered usually exhibit T2 hyperintensity, with a various enhancement pattern. In our study, the only one case of DMG exhibited ring-like enhancement. In diffuse pediatric-type high-grade gliomas, H3-wildtype and IDH-wildtype, peritumoral edema and intratumoral necrosis, which are common characteristics of high-grade gliomas, were mild but frequently observed. Notably, there was an heterogeneity observed in imaging characteristics of the 7 diffuse pediatric-type high-grade gliomas, H3-wildtype and IDH-wildtype in our study. As previously mentioned, diffuse pediatric-type high-grade glioma, H3-wildtype and IDH-wildtype can be further classified into 3 subtypes based on DNA methylation characteristics, and this imaging heterogeneity may be explained by the fact that different subtypes have different imaging patterns. For example, it has been reported that MYCN may be better circumscribed, with only mild peritumoral edema and homogeneous contrast enhancement (Tauziède-Espariat et al., 2020). The imaging characteristics of the other 2 subtypes, RTK1 and RTK2, have not been reported. Although further case accumulation is needed to draw a definitive conclusion, this imaging heterogeneity could be a future direction of research.

As a member of histone lysine methyltransferases (KMTs), *KMT5B* converts H4K20me1 into H4K20me2 and functions in a tumor suppressor-like manner. Through epigenetic silencing of oncogenes such as *IL13RA2*, overexpression of *KMT5B* reduced glioblastoma cell proliferation, cell viability, clonogenic potential *in vitro*, and tumor growth *in vivo* (López et al., 2021; Hulen et al., 2022). Two inactivating mutations of *KMT5B* (R187* and R699*) were identified in glioblastoma and diffuse intrinsic pontine glioma (DIPG) samples, which abrogated DNA repair and increased invasion and migration in neighboring cells (Vinci et al., 2018). Notably, 5 of 6 patients with altered *KMT5B* in our study had the same mutation of p.Glu833_Asp835delinsSerProSer, including 3 diffuse astrocytomas, *MYB*- or *MYBL1*-altered and 2 diffuse pediatric-type high-grade gliomas, H3-wildtype and IDH-wildtype. Considering their significantly shorter overall survivals and the anti-tumoral effect of *KMT5B*, it could be suspected that this deletion–insertion in the C-terminal region may cause functional defect to *KMT5B* and de-repression of oncogenes in a subgroup of adult patients with pediatric-type glioma. At the same time, the underlying mechanisms guarantee further investigation on a molecular level.

*MET* is among the top three dysregulated receptor tyrosine kinases (RTKs) in glioma cells, along with *EGFR* and *PDGFRA* (Snuderl et al., 2011). Activating mutations in *MET* are key events during the progression of low-grade gliomas to a higher grade, while *MET* gain in diffuse astrocytoma is associated with shorter overall survival (Pierscianek et al., 2013; Hu et al., 2018). Therefore, our finding that adult pediatric-type glioma patients with *MET* alterations have worse prognoses could be supported by previous results. In our study, 2 patients with diffuse pediatric-type high-grade glioma, H3-wildtype and IDH-wildtype and 1 patient with diffuse midline glioma, H3 K27-altered had *MET* alterations. *MET* amplification is a common (22.9%) alteration in diffuse pediatric-type high-grade glioma, H3-wildtype and IDH-wildtype (Hong et al., 2022). In diffuse midline glioma, H3 K27-altered, *MET* alterations including amplification and activating point mutations have been reported to occur sporadically (8.6–15.0%) (Hoffman et al., 2016; Porkholm et al., 2018; Dufour et al., 2020). No correlation between *MET* status and survival has been discovered in either of these two pediatric-type high-grade glioma subtypes (Hoffman et al., 2016; Porkholm et al., 2018; Dufour et al., 2020; Hong et al., 2022). Consistent with previous literature (Fabbri et al., 2022; Kalelioglu et al., 2022), *MET* alterations were not detected in any of our diffuse astrocytoma, *MYB*- or *MYBL1*-altered patients. *MET*-altered patients may merit particular attention in clinical management, especially in tumor recurrence. Bevacizumab has now become a common practice for recurrent glioma patients. However, inhibiting *VEGFA* could negate its suppression of *HGF*-dependent *MET* phosphorylation and tumor cell migration and lead to a more invasive phenotype (Lu et al., 2012). In light of these findings, combining anti-*VEGFA* and anti-*MET* treatments might clinically benefit *MET*-altered adult patients with pediatric glioma.

## Limitations

Our study results must be interpreted while considering some limitations. First of all, the systematic bias raised by the small sample size should be taken into consideration. In addition, forms of molecular alteration are various, in which simply classifying the molecular features as normal and altered may cover up some significant changes. Also, methylation profiling analysis was not conducted in the molecular diagnoses. Therefore, diagnostic uncertainties might exist. Other sources of errors include systematic errors produced during the process of immunohistochemical staining, data measurement, and observation (including imaging and patient follow-up). Therefore, further studies are necessary to confirm these findings.

## Future developments

In this study, we made a summative description and preliminary analysis of adult patients with pediatric diffuse glioma in light of the novel 2021 WHO classification of CNS tumors and suggested the correlation between molecular features and patients’ prognosis. Our research aims to expand the current understanding of adult patients with pediatric-type diffuse gliomas and to complement the limited research. Furthermore, personalized therapies consisting of targeted molecular inhibitors for *MET* and *VEGFA* may exhibit beneficial effects in the corresponding population.

## Data availability statement

The original contributions presented in the study are included in the article/Supplementary material, further inquiries can be directed to the corresponding authors.

## Ethics statement

The studies involving human participants were reviewed and approved by the Institutional Ethics Review Board of PUMCH. Written informed consent for participation was not required for this study in accordance with the national legislation and the institutional requirements.

## Author contributions

WC and XG: study conception and design. WC, SJ, QL, HW, YX, XG, SG, YNW, YS, DL, YL, YKW, HX, JL, JW, TL, TQ, HL, TY, and KZ: data collection. WC, SJ, QL, HW, and YX: data analysis and figures and tables. WC, SJ, QL, HW, YX, and XG: manuscript drafting and revision. YW and WM: study supervision. All authors contributed to the article and approved the submitted version.

## Funding

This work was funded by the Beijing Municipal Natural Science Foundation (7202150) and the National High-Level Hospital Clinical Research Funding (2022-PUMCH-A-019) for YW and by the National High-Level Hospital Clinical Research Funding (2022-PUMCH-B-113), the Tsinghua University-Peking Union Medical College Hospital Initiative Scientific Research Program (2019ZLH101), and the Beijing Municipal Natural Science Foundation (19JCZDJC64200[Z]) for WM.

## Conflict of interest

The authors declare that the research was conducted in the absence of any commercial or financial relationships that could be construed as a potential conflict of interest.

## Publisher’s note

All claims expressed in this article are solely those of the authors and do not necessarily represent those of their affiliated organizations, or those of the publisher, the editors and the reviewers. Any product that may be evaluated in this article, or claim that may be made by its manufacturer, is not guaranteed or endorsed by the publisher.
