# Supplementary material for: Spotlights on adult patients with pediatric-type diffuse gliomas in accordance with the 2021 WHO classification of CNS tumors
Source: Front Neurosci. 2023 May 5;17:1144559. doi: 10.3389/fnins.2023.1144559 (PMC10196618; doi:10.3389/fnins.2023.1144559)
Supplement: Supplementary file 1 [file Table_1.docx]

| List of molecular markers. | | | | | | | | | |
| --- | --- | --- | --- | --- | --- | --- | --- | --- | --- |
| *ACVR1* | *ATRX* | *BCOR* | *BRAF* | *CDK4* | *CDK6* | *CDKN2A* | *CDKN2B* | chr10p | chr10q |
| chr17 | chr19q | chr1p | chr7p | chr7q | chr9p | *CIC* | *EGFR* | *FBXW7* | *FGFR1* |
| *FGFR2* | *FGFR3* | *FGFR4* | *FUBP1* | *H3F3A* | *HIST1H3B* | *HIST1H3C* | *IDH1* | *IDH2* | *KIT* |
| *KMT5B* | *KRAS* | *MAP2K1* | *MET* | *MYB* | *MYBL1* | *MYC* | *MYCN* | *NF1* | *NOTCH1* |
| *NRAS* | *NTRK2* | *NTRK3* | *PDGFRA* | *PEG3* | *PIK3CA* | *PIK3CB* | *PIK3R1* | *PPM1D* | *PTEN* |
| *PTPN11* | *RB1* | *SMARCA4* | *SMARCB1* | *TERT* | *TOP3A* | *TP53* | *TSC1* | *TSC2* | *YAP1* |

Supplementary Table 1. The complete list of molecular markers.
