# Supplementary figures and images for: Spotlights on adult patients with pediatric-type diffuse gliomas in accordance with the 2021 WHO classification of CNS tumors
Source: Front Neurosci. 2023 May 5;17:1144559. doi: 10.3389/fnins.2023.1144559 (PMC10196618; doi:10.3389/fnins.2023.1144559)

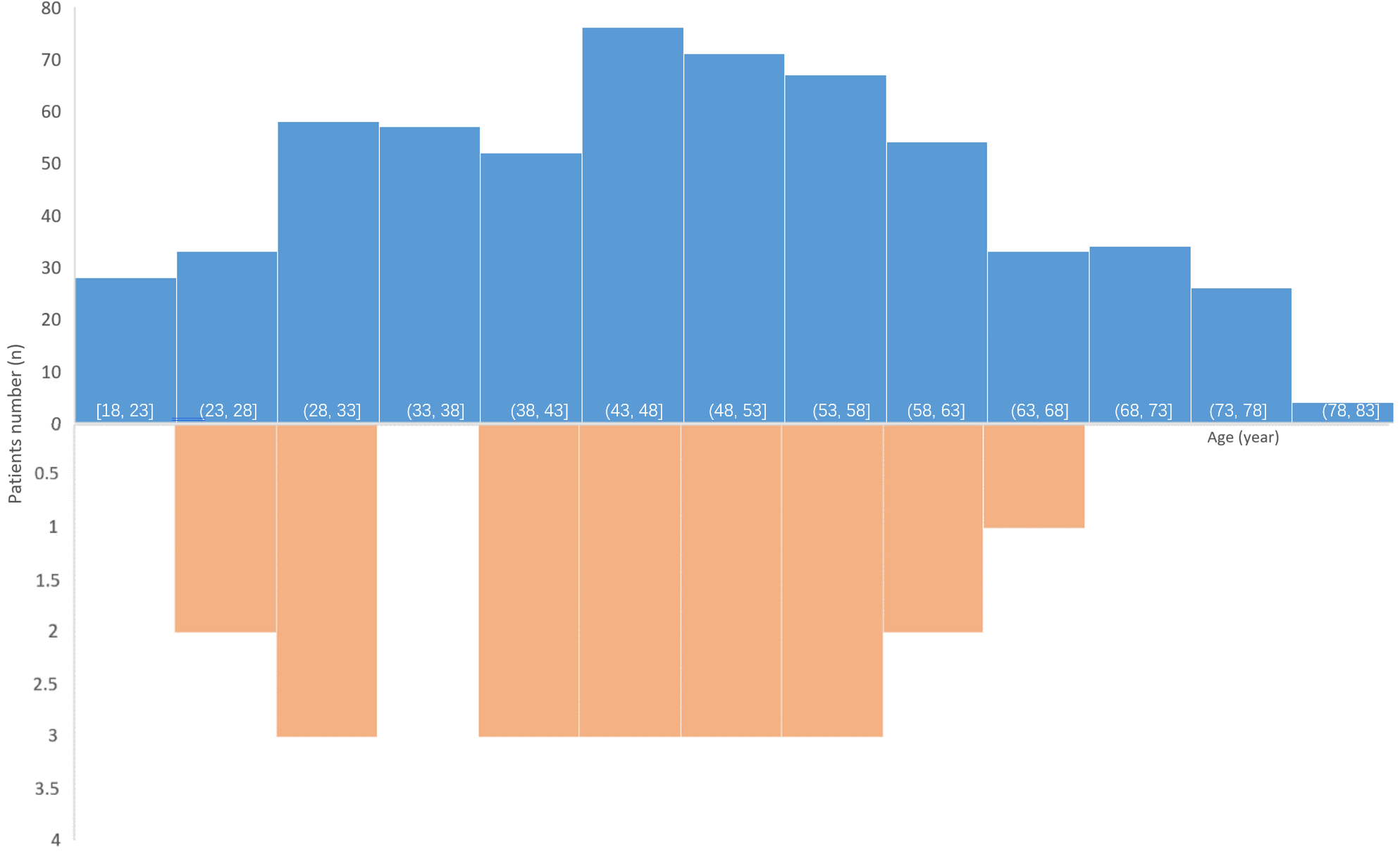

Supplement: Supplementary file 2 [file Image_1.png]
